# A fast and reliable method for monitoring of prophage‐activating chemicals

**DOI:** 10.1111/1751-7915.13042

**Published:** 2018-01-12

**Authors:** Juan Xu, Bärbel Kiesel, René Kallies, Feng‐Lei Jiang, Yi Liu, Thomas Maskow

**Affiliations:** ^1^ State Key Laboratory of Virology College of Chemistry and Molecule Sciences Wuhan University Wuhan 430072 China; ^2^ Department of Environmental Microbiology UFZ – Helmholtz Centre for Environmental Research Permoserstrasse 15 04318 Leipzig Germany; ^3^ Key Laboratory of Analytical Chemistry for Biology and Medicine (Ministry of Education) College of Chemistry and Molecule Sciences Wuhan University Wuhan 430072 China

## Abstract

Bacteriophages, that is viruses that infect bacteria, either lyse bacteria directly or integrate their genome into the bacterial genome as so‐called prophages, where they remain at a silent state. Both phages and bacteria are able to survive in this state. However, prophages can be reactivated with the introduction of chemicals, followed by the release of a high number of phage particles, which could infect other bacteria, thus harming ecosystems by a viral bloom. The basics for a fast, automatable analytical method for the detection of prophage‐activating chemicals are developed and successfully tested here. The method exploits the differences in metabolic heat produced by *Escherichia coli* with (λ+) and without the lambda prophages (λ−). Since the metabolic heat primarily reflects opposing effects (i.e. the reduction of heat‐producing cells by lysis and enhanced heat production to deliver the energetic costs for the synthesis of phages), a systematic analysis of the influence of the different conditions (experimentally and *in silico*) was performed and revealed anoxic conditions to be best suited. The main advantages of the suggested monitoring method are not only the possibility of obtaining fast results (after only few hours), but also the option for automation, the low workload (requires only few minutes) and the suitability of using commercially available instruments. The future challenge following this proof of principle is the development of thermal transducers which allow for the electronic subtraction of the λ+ from the λ‐ signal.

## Introduction

Bacteriophages, or phages, are viruses which infect bacteria. Phages play important roles in different ecosystems. They influence biogeochemical cycles (e.g. by the release of nutrients of lysed bacteria), and as a result, they are able to shape the composition and structure of the microbial community (Hambly and Suttle, 2005; Paul, 2008; Thingstad *et al*., 2008; Rohwer and Thurber, 2009; Richter *et al*., 2012; Aziz *et al*., 2015; Howard‐Varona *et al*., 2017). Phages can multiply in their hosts by two different life cycle strategies. The lytic cycle, used by lytic (or virulent) phages, is characterized by the lysis of the bacterial–host cell after infection. In contrast, the temperate phages integrate their genome into the host cell chromosome, and their DNA is replicated along with the host cell DNA (lysogenic cycle). The integrated phage genome is termed prophage. This prophage can silently coexist with its host until it becomes activated by different environmental influences, e.g. UV irradiation, changes of pH, temperature and water activity (Schmidt, 2001; Wagner and Waldor, 2002; Herold *et al*., 2004). In addition, anthropogenic chemicals are known to activate prophages (Motlagh *et al*., 2015). A common feature of prophage activation is the induction of the host SOS system, which is accompanied by an irreversible conversion of lysogeny to lysis. Hence, the phages are released into the environment and are able to infect further bacteria, which might result in shifts in community composition and a reduced abundance of affected host species (Rohwer and Thurber, 2009). When considering the short time between prophage activation and host cell lysis (30–90 min) as well as the high number of new phage particles possibly being released into the environment (tens to a few hundred phages per bacterial cells), prophages might become a ‘dangerous molecular time bomb’ (Paul, 2008). Many carcinogenic agents (Elespuru and White, 1983), antibiotic substances (Tanouchi *et al*., 2013; Bearson and Brunelle, 2015), organic pollutants (Cochran *et al*., 1998), emerging micropollutants, such as personal care products (Danovaro and Corinaldesi, 2003) and pharmaceuticals, or even mixtures from industrial wastes (Houk and DeMarini, 1988) are among the chemicals that are known to activate prophages. An important requirement in assessing chemicals in terms of their potential to activate prophages is the quantitative determination of phage production kinetics after prophage activation. This can be achieved by either counting released phage particles over time or by direct online monitoring of the phage production process. Many different methods for phage particle counting are developed over the last decades (Adams, 1950; Shibata *et al*., 2006). The double agar overlay assay is usually considered to be the gold standard for phage quantification (Adams, 1950) for which a phage‐sensitive bacterial indicator strain is necessary. Another limitation with this assay is that only native phages can be counted as plaque‐forming units (*pfu*), nevertheless with a large error rate (Abedon and Yin, 2009). Another method of phage counting is microscopy, either with or without labelling (Zago *et al*., 2012; Alsteens *et al*., 2013). An advantage of microscopy over the other techniques is the possibility of determining infectious as well as non‐infectious phage particles. This is however very laborious and requires a high number of phage particles. Further methods to quantify phages are: (i) quantitative (real‐time) PCR (Waller *et al*., 2014) (Refardt, 2012), (ii) spectrophotometry of the major coat protein VIII (Smith, 1997), (iii) Raman spectroscopy (Goeller and Rilley, 2007), as well as (iv) pulsed‐field gel electrophoresis and LASER densitometry (Klieve and Swain, 1993). Thorough sample purification along with a specific pre‐treatment prior to the analysis are prerequisites for all of these methods, making them rather less suitable for fast high‐throughput analysis and requiring highly qualified personnel. A sophisticated method that solves the high‐throughput problem and promises short detection times is based on electric chips, for instance, for DNA (Gabig‐Ciminska *et al*., 2004). Here is the main problem the development and cost‐intensive production of bead immobilized capture probes and the requirement of sample preparation.

In contrast, calorimetry is a non‐invasive, non‐destructive, real‐time method that allows the direct monitoring of the phage production after the chemically induced activation of prophages. This has recently been demonstrated for a fermentation calorimeter (Maskow *et al*., 2010). However, fermentation calorimeters can only be used for the analysis of a few samples and are therefore not appropriate for high‐throughput analyses. In other studies, a multichannel isothermal microcalorimeter has been used to monitor the lysis of *E. coli* cells after T4 prophage induction (Liu *et al*., 2003, 2005). Chip‐calorimetric techniques have also proven to be useful in monitoring phage production (Lerchner *et al*., 2011; Mariana Morais *et al*., 2014) and were proposed for high‐throughput measurements using chip‐arrays or segmented flow technology (Wolf *et al*., 2015). This lab‐on‐chip technology is yet still under development, with challenges in mixing on a very small scale. Considering these circumstances, a simple and reliable technology is needed for testing the properties of prophage‐inducing chemicals. Therefore, a proof of concept has been developed based on the use of a thermal transducer, which is not only suitable for automation and high‐throughput measurements, but also allows a simple data interpretation.

## Results and discussion

### Measuring principle

**Figure 1:**
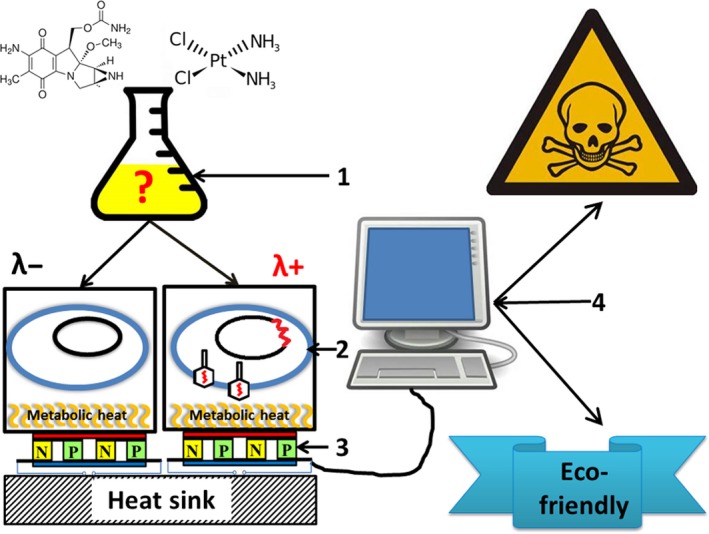
Measurement principle: 1: preparation of the chemical or of the mixture to be examined; 2: λ− and λ+ strains as the bioindicators; 3: thermal transducers (Peltier elements); 4: electronic evaluation unit.

The measuring principle of the cell‐based biosensor is sketched in Fig. 1. Peltier elements are used to measure continuously the difference in the metabolic heat production of the λ+ and λ‐ bioindicator strains. When a predefined difference is exceeded, the chemical can be considered as dangerous (due to prophage activation). The usage of the voltage difference between the two Peltier elements has the advantage that either potential physical distortions (e.g. temperature) or biological distortions (e.g. other toxicity targets of the chemical, or the respective physiological state of the bioindicator) have only minimal influence on the signal to be detected, as both sides are equally affected. Since multichannel instruments that allow for the direct subtraction of the Peltier signals are not available, the λ+ and λ− strains were measured independently for testing the cell‐based biosensor principle and the subsequent comparison of the obtained results. Not only minor variations during the preparation but also slight differences in the physiological state of the bioindicators cannot be excluded, as both could influence the final outcome of this experimental approach.

### Metabolic factors affecting the heat signal

**Figure 2:**
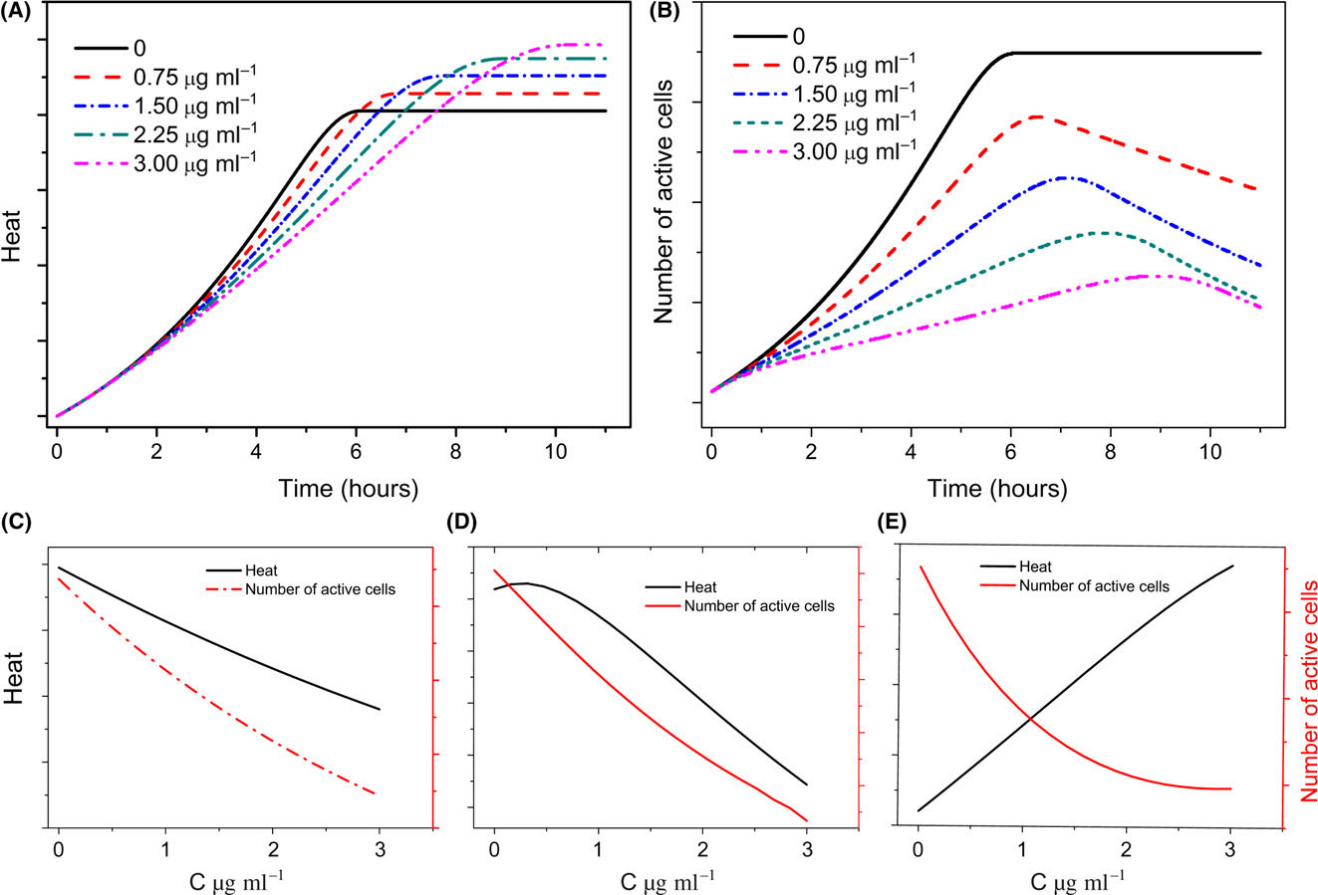
Expected effects of the prophage‐activating chemical on heat (A) and the number of active cells (B). Comparison of the influence of the phage‐activating chemical on heat and the number of active cells after different incubation times of the bioindicator (C: 4 h; D: 6 h; F: 11 h). The details of the simulation model and of the used parameters are given in Supporting Information.

Two counteracting effects (i.e. growth inhibition and phage production) may be assumed after prophage activation. First, the growth rate of the host cell decreases (lysis of cells). As a result, the metabolic heat of the bioindicator culture and the dosage of the inducing chemical should be inversely proportional. Second, as reported previously (Liu *et al*., 2003, 2005; Mariana Morais *et al*., 2014), the energetic cost of building a virus (phage) (Mahmoudabadi *et al*., 2017) results in an elevated cell‐specific heat production rate. The consequence is an opposite effect of a direct proportionality between the metabolic heat and the dosage. Therefore, we should expect a clear influence when one of the effects is dominating. Mathematical modelling was used to simulate these different conditions (for details, see Supporting Information). The results for a model system with typical *E. coli* growth parameter are shown in Fig. 2.

As expected, the simulation shows a clear dosage dependency between the number of cells and the concentration of the test chemical (Fig. 2B). The predicted maximum is determined by growth, on the one side, and the reduction in cell numbers by the entry in the lytic phase, on the other. Even in the case of metabolic heat, a dosage dependency is obvious (Fig. 2A). Surprisingly, dosage dependency to changes in heat over the incubation time was detected (Fig. 2C–E). Two completely different situations are predicted at the beginning of the chemical incubation and after the consumption of the substrate. At the beginning, the influence of the test chemical on the number of active cells and heat is similar (Fig. 2C). In contrast, at the end the influence of the test chemical on the number of active cells is opposite to the heat (Fig. 2E). For the cell‐based biosensor development, a fast detection time is important. Therefore, the focus of the biosensor development is on the evaluation of the initial signal. In this measuring period, it is important to minimize the residual influence of heat from phage production on the total signal. Of course, our simple simulation provides some useful thesis for the cell‐based biosensor behaviour, but it only reflects the assumed main effects and predictions that have to be considered with care and further tested experimentally.

### Influence of oxygen

The energetic costs of building a phage are provided by the catabolism. The maximum possible catabolic energy gain depends on the electron donor/acceptor couple. From the technical perspective, air oxygen is the easiest available terminal electron acceptor. Therefore, in the following, the influence of oxygen bioavailability on the bioindicator is analysed. In the simplest case, the bioindicator is suspended in liquid medium and subsequently the obtained suspension is exposed to air oxygen. Under these conditions, the prophage activation could be confirmed by the reference methods (i.e. the cell number and pfu), but not unambiguous by the metabolic heat (see Supporting Information, Figs S1 and S2). A metabolic shift from respiratory to respiro‐fermentative growth (Maskow *et al*., 2014) could be speculated as a potential reason for this ambiguous result. The metabolic shift is a result of the antagonistic effects of a slow oxygen delivery by diffusion and fast oxygen consumption by growth.

The growth of the bioindicator on the agar surface (with short diffusion ways and high gas diffusion coefficients) could diminish this potential error source. The results of growth on agar surfaces are exemplarily shown in the Supporting Information (Fig. S3). Although prophage activation has been confirmed by *pfu* counting, the heat trace cannot be unambiguously associated with the phage production.

**Figure 3:**
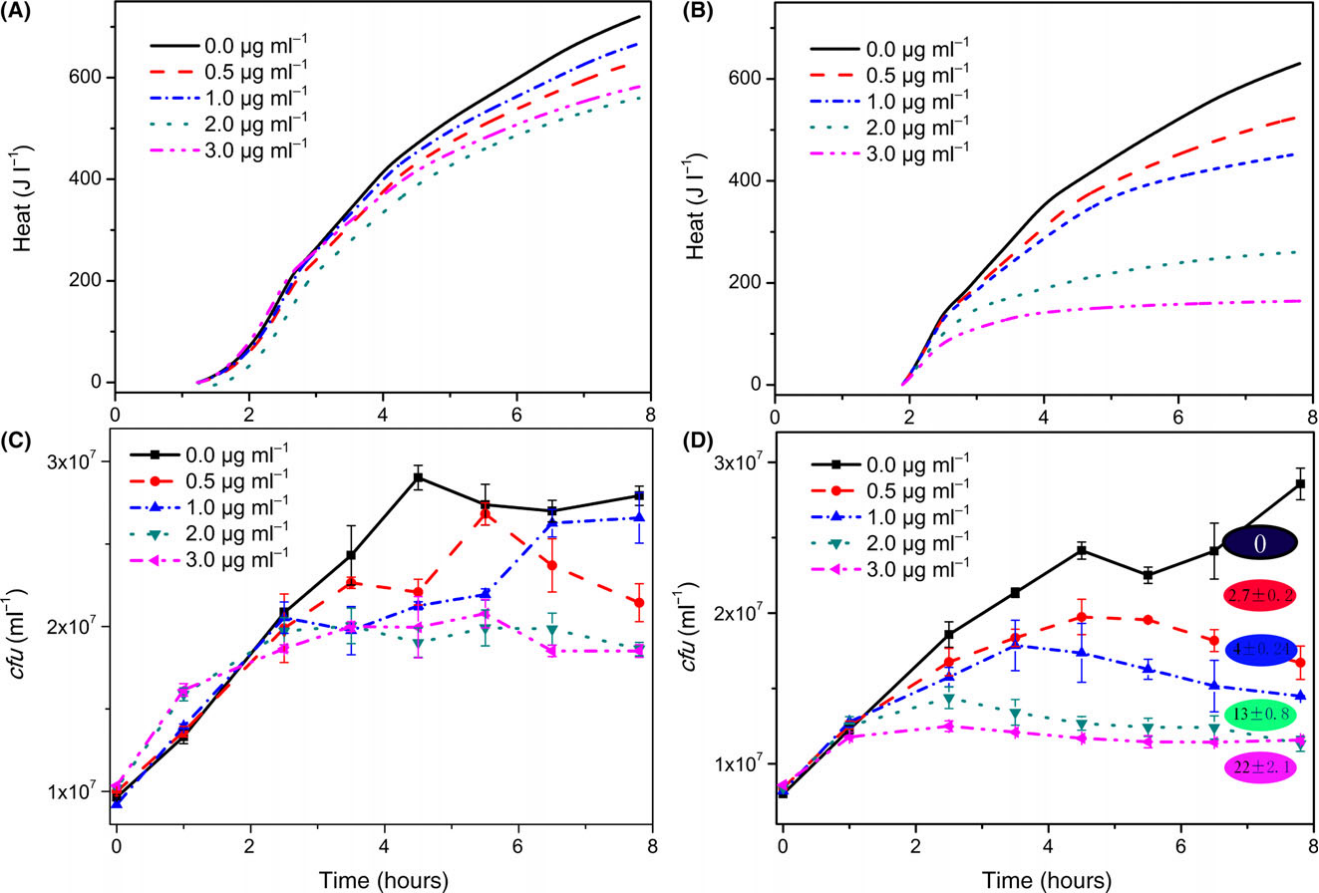
Behaviour of the bioindicator (λ− left‐hand side; λ+ right‐hand side) in the presence of different amounts of mitomycin C. A and B show the heat traces of the bioindicator. C and D indicate the independently measured bioindicator growth. The final phage numbers (given as *pfu* after 8 h) are included in circles (*10^7^ ml^−1^) in D.

The last remaining technically simple way to influence the catabolic energy production is the exclusion of oxygen. This can be achieved using a thin layer of metabolically inert oil on the surface of a bacterial suspension. It finally led to cell‐based biosensor heat signals that work. Here, the difference between λ+ and λ− is strongly dosage dependent (Fig. 3A and B). The heat signal as measure for prophage activation is confirmed by a difference in cell numbers (Fig. 3C and D) as well as by differences in phage release by *pfu* determinations after 8 h.

In case of anaerobic growth, the effect of the cell reduction clearly dominates the energetic effect of phage production. Taking the substrate glucose as an example, the aerobic catabolism dissipates −2813.6 kJ mol^−1^ as heat and produces approximately 26 mol ATP mol^−1^ (assuming a P/O = 2), whereas the anaerobic metabolism dissipates −100 kJ mol^−1^ (von Stockar *et al*., 1993) and produces 2 mol ATP mol^−1^ (Embden Meyerhof glycolytic pathway). When assuming the same ATP requirement for phage synthesis, twice as much of the heat is generated under aerobic conditions compared with anaerobic conditions. It follows from these considerations that the combination of anaerobic growing bioindicators with thermal transducers let general expects the highest effects.

### Test of the method with different prophage‐activating chemicals

**Figure 4:**
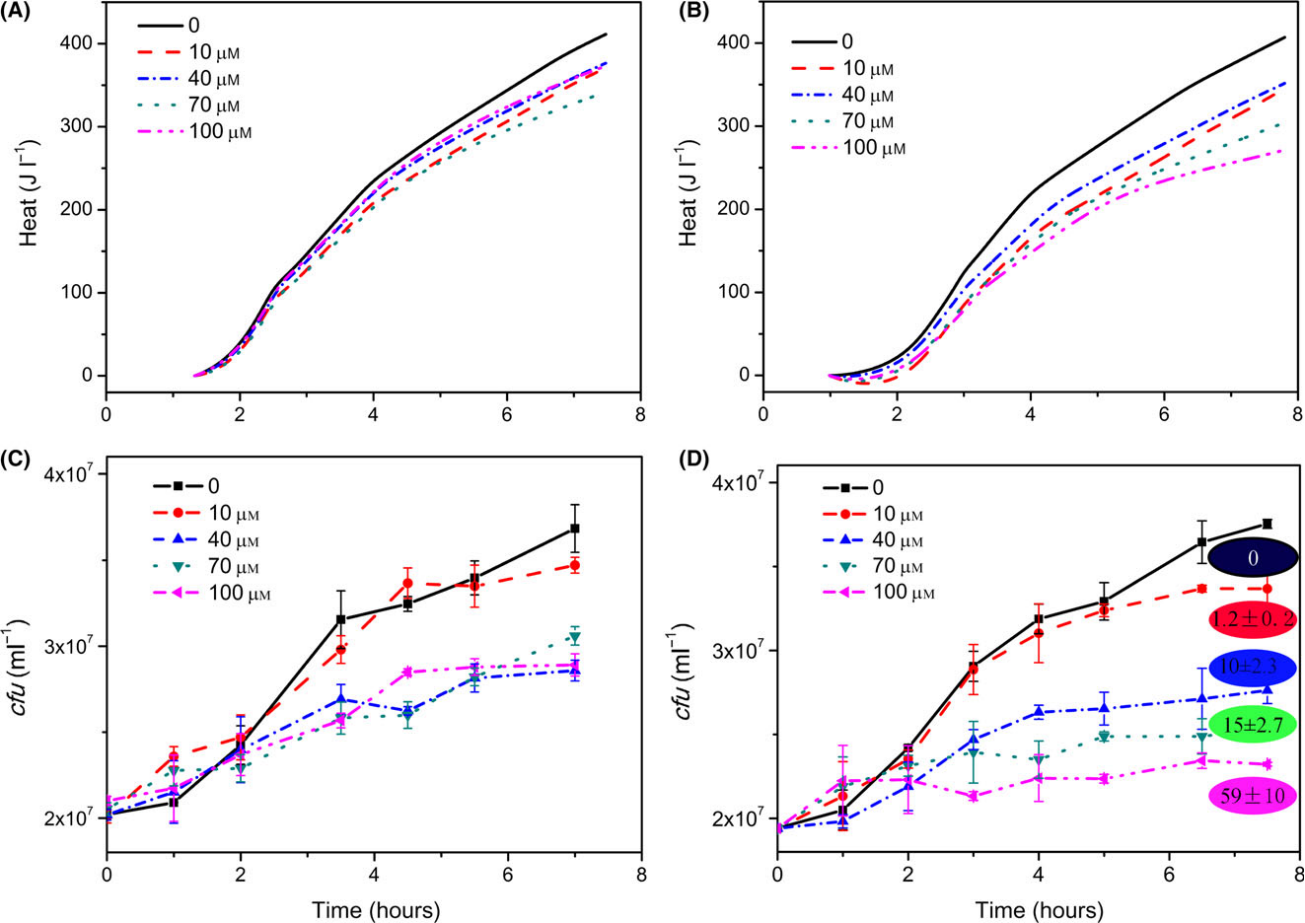
Behaviour of the bioindicator (λ− left‐hand side; λ+ right‐hand side) in the presence of different amounts of Cis‐platinum without oxygen. A and B illustrate the heat traces of the bioindicator, whereas C and D represent the independently measured growth of the bioindicator. The final phage numbers (given as *pfu* after 8 h) are included in circles (*10^5^ ml^−1^) in D.

In the following, the bioindicator behaviour will be evaluated with two further prophage‐activating chemicals. Even inorganic materials are known to activate silent prophages (Rossman *et al*., 1984; Houk and DeMarini, 1988). Therefore, the bioindicator has to be tested by using examples of inorganic chemicals. *Cis*‐platinum is perfect for that purpose because it has already been proven to induce prophages (Mattern *et al*., 1982), and the mechanism is well known. As the comparison of Fig. 4A and B shows, the biosensor is properly working even for inorganic chemicals. A clear dosage dependency of the heat traces was found. The result is confirmed by the cell numbers (Fig. 4C and D) as well as by the *pfu* after 8 h.

**Figure 5:**
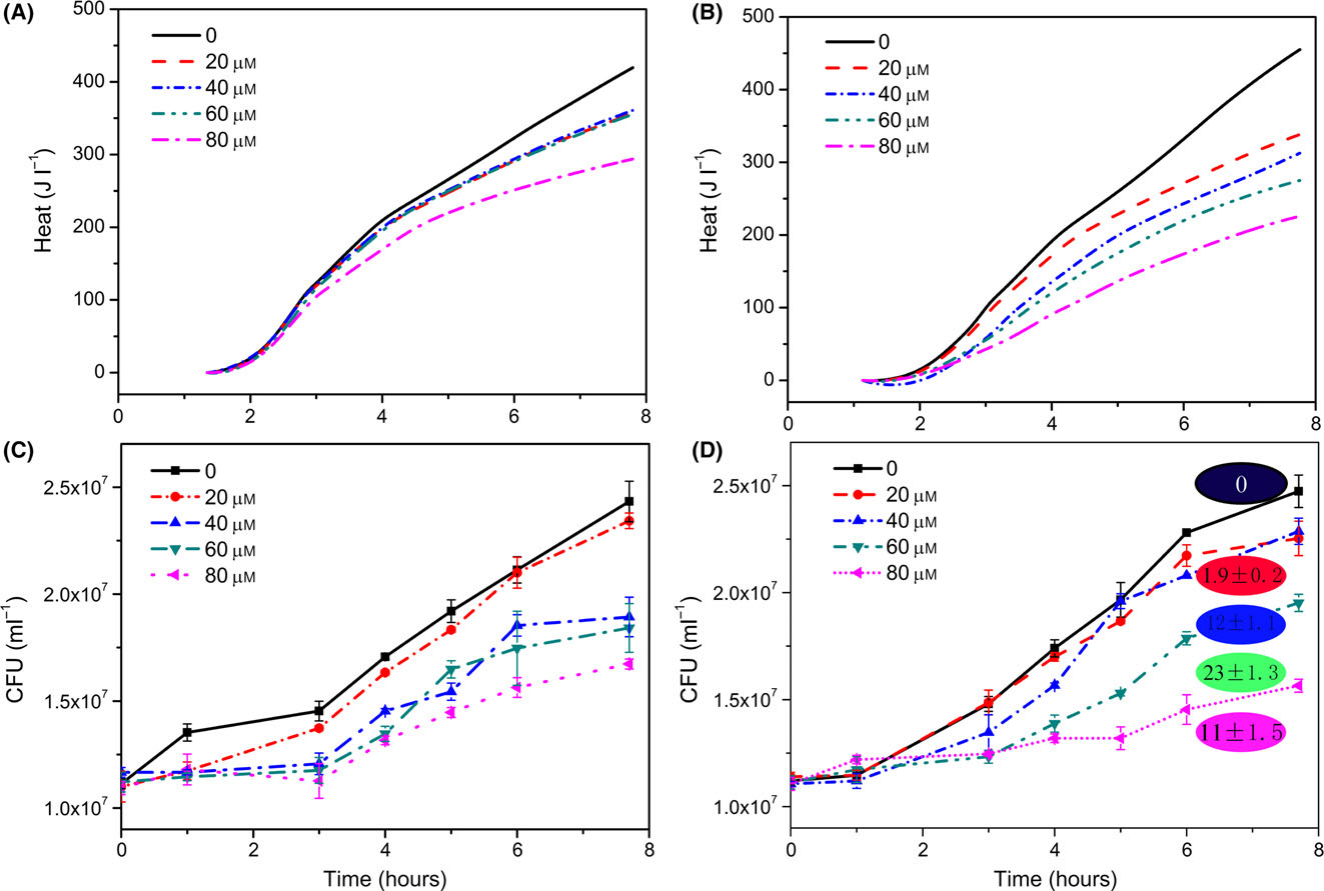
Behaviour of the bioindicator (λ− left‐hand side; λ+ right‐hand side) in the presence of various amounts of different chromium (6) concentrations without oxygen. A and B show the heat traces of the bioindicator, and C and D demonstrate the independently measured bioindicator growth. The final phage numbers (given as *pfu* after 8 h) are included in circles (*10^5^ ml^−1^) in D.

The results for the test chemical of hexavalent chromium are exemplarily shown in Fig. 5. The proper working of the cell‐based biosensor for testing prophage‐activating properties of chemicals is again confirmed.

## Conclusion

In summary, it can be concluded that combining bioindicators λ+ and λ‐ with a thermal transducer and subsequently considering the signal difference is suited in principle to screen for prophage‐activating properties of chemicals. The thermal transducer has the advantage to reflect the total process of prophage activation and phage production. However, the oxygen has to be excluded from the bioindicator. The motives are overlapping and opposite‐acting effects. The chemical leads to a shift in the lytic cycle that reduces the number of heat‐producing bacteria, on one side, whereas the infected bacteria produce more heat than the uninfected ones, on the other. This difference in heat production might be reduced by operating the cell‐based biosensor under anaerobic conditions.

After our proof of concept, we see two main approaches to improve the sensitivity of the biosensor. The first approach is the development of a transducer, which allows the electronic subtraction of the Peltier signal of the λ+ from the respective signal of the λ− strain. As a result, the influence of small variations (in environmental temperature, physiological state of the bioindicator, concentration of the sensing bacteria, etc.) on the signal could be reduced. The second approach for improving the biosensor is the substitution of the λ+ side by a mixture of λ+ with λ− strains. Thereby, it is expected that the phages produced by activated λ+ strains would infect the λ− strains. As a result, the thermal effect would be amplified.

The potential of our suggested cell‐based biosensor for future high‐throughput measurements is supported (i) by the development of multichannel calorimetric instruments(see e.g. Wadsö *et al*., 2017), (ii) by the development of arrays of chip‐calorimeters (Torres *et al*., 2010; Huynh *et al*., 2015), and (iii) by the segmented fluid technology for a chip‐calorimeter.(Maskow *et al*., 2011; Wolf *et al*., 2015) Future research should aim to open up additional application areas. As the prophage activation can be considered as a measure of genotoxicity and the mutagenic properties of chemicals (Moreau *et al*., 1976; Elespuru and White, 1983) the proposed cell‐based biosensor is even applicable in medicine. This holds not only true for pure chemicals, but also for mixtures such as plant or fungi extracts (Taghvaei *et al*., 2009).

## Experimental procedures

*Escherichia coli* strains (DSM 4230) with (λ+) and without prophage (λ−) were used as the bioindicator. Details about the generation of the strains, the cultivation and the conditions during biosensing are given in the Supporting Information. For the development and testing of the cell‐based biosensor, Mitomycin C (Otsuji *et al*., 1959; Yamamoto and Chow, 1968) was selected as an example for an organic inducer, whereas Cis‐platinum ((SP‐4‐2)‐diamminedichloroplatinum(II)) and hexavalent chromium (chromate) served as examples for inorganic inducers. All test substances are well‐known prophage‐activating chemicals. The Thermal Activity Monitor III (TAM III; TA Instruments, New Castle, DE, USA) was used as a thermal transducer. The ampoules and caps were autoclaved (30 min, 121°C) prior to the experiments. Electric gain calibrations were regularly performed. The calorimetric vessels were filled with 1.9 ml LB medium (containing different concentrations of the respective prophage‐inducing chemicals) and 0.1 ml bacterial suspension (OD = 0.1 in LB medium). Anaerobic experiments were performed with the addition of 0.2 ml of liquid paraffin to prevent oxygen diffusion in the medium. Any traces of oxygen that may be present in the medium are consumed in a few minutes due to the high bacterial density. Therefore, one can consider the growth as anaerobic. The determination of both the optical density and the plaque‐forming units was used as the reference methods due to simplicity of use. A calibration curve enabled us to relate the optical density to the corresponding cell number. The optical density was measured at 600 nm (UV‐Vis‐100PC; Hitachi High‐Tech, Tokyo, Japan). The numbers of induced phages were quantified as plaque‐forming units (*pfu*) per ml by means of the double‐layer technique according to Adams (1950). Mathematical simulations were applied to understand the influence of various factors on both the behaviour of the bioindicator and the respective heat signal. The model for the simulation was created by using the berkley madonna Version 8.1, developed by R.I. Macey and G. F. Oster at the University of California. The details of the simulation (the biological and physical background, describing differential equations, numerical integration and detailed results) are described in the Supporting Information.

## Conflict of interest

None declared.

## Supporting information


**Appendix S1.** Experimental conditions, mathematical modeling of the cell‐based biosensor behavior and the optimization of the biosensor.
**Fig. S1.** PCR based integration profiles of the lambda prophage. M denotes the size marker with a 1000 bp and 500 bp dense band; (1) negative control without a template, (2) *E. coli* DSM 4230 WT without a prophage, (3) *E. coli* K124 (lambda) as a positive control, (4)(5)(6)(7) are lambda lysogenic isolates of *E. coli* DSM4230: Isolates (4)(6)(7) are multilysogenic, and isolate (5) is the single lysogenic strain *E. coli* DSM4230 (lambda)‐47.
**Fig. S2.** The behavior of the bioindicator (λ− left hand side; λ+ right hand side) in the presence of different amounts of Mitomycin C. A and B show the heat traces and C and D denote the growth traces. The final phage numbers (*10^6^ ml^‐1^ given as *pfu* after 8 h) are included in D.
**Fig. S3.** Behavior of the bioindicator (λ− left hand side; λ+ right hand side) in the presence of different amounts of *cis*‐platinum C. A and B show the heat traces, and C and D demonstrate the growth traces. The final phage numbers (*10^4^ ml^‐1^ given as PFU after 8 h) are included in D.
**Fig. S4.** Behavior of the bioindicator (λ− left hand side; λ+ right hand side) grown on agar in the presence of different amounts of Mitomycin C. The final phage numbers (*10^6^ ml^‐1^ given as *pfu* after 24 h) are included in circles.
