# Supplementary material for: A fast and reliable method for monitoring of prophage‐activating chemicals
Source: Microb Biotechnol. 2018 Jan 12;11(6):1112–20. doi: 10.1111/1751-7915.13042 (PMC6196395; doi:10.1111/1751-7915.13042)
Supplement: Supplementary file 1 — Appendix S1. Experimental conditions, mathematical modeling of the cell‐based biosensor behavior and the optimization of the biosensor. Fig. S1. PCR based integration profiles of the lambda prophage. M denotes the size marker with a 1000 bp and 500 bp dense band; (1) negative control without a template, (2) E. coli DSM 4230 WT without a prophage, (3) E. coli K124 (lambda) as a positive control, (4)(5)(6)(7) are lambda lysogenic isolates of E. coli DSM4230: Isolates (4)(6)(7) are multilysogenic, and isolate (5) is the single lysogenic strain E. coli DSM4230 (lambda)‐47. Fig. S2. The behavior of the bioindicator (λ− left hand side; λ+ right hand side) in the presence of different amounts of Mitomycin C. A and B show the heat traces and C and D denote the growth traces. The final phage numbers (*106 ml‐1 given as pfu after 8 h) are included in D. Fig. S3. Behavior of the bioindicator (λ− left hand side; λ+ right hand side) in the presence of different amounts of cis‐platinum C. A and B show the heat traces, and C and D demonstrate the growth traces. The final phage numbers (*104 ml‐1 given as PFU after 8 h) are included in D. Fig. S4. Behavior of the bioindicator (λ− left hand side; λ+ right hand side) grown on agar in the presence of different amounts of Mitomycin C. The final phage numbers (*106 ml‐1 given as pfu after 24 h) are included in circles. [file MBT2-11-1112-s001.docx]

**Supplementary Material**

**“A Fast and Reliable Method for Monitoring of Prophage Activating Chemicals”**

**Juan Xu^a,c^, Bärbel Kiesel^b^, René Kallies^b^, Feng-Lei Jiang^a,c^, Yi Liu^a,c^, Thomas Maskow^b^**

^a^ State Key Laboratory of Virology, College of Chemistry and Molecule Sciences, Wuhan University, Wuhan 430072, P. R. China

^b^ UFZ – Helmholtz Centre for Environmental Research, Department of Environmental Microbiology, Permoserstrasse 15, 04318 Leipzig, Germany

^c^ Key Laboratory of Analytical Chemistry for Biology and Medicine (Ministry of Education), College of Chemistry and Molecule Sciences, Wuhan University, Wuhan 430072, P. R. China

**Corresponding authors:**

PD Dr. Thomas Maskow; e-mail: Thomas.maskow@ufz.de; Phone: +49 341 235 1328

Prof. Yi Liu; e-mail: yiliuchem@whu.edu.cn; Phone: +86-18971489606

Contents

[1. *In-silico* experiments 2](#_Toc498673949)

[i) Bioindicator behavior 2](#_Toc498673950)

[ii) Limiting conditions 2](#_Toc498673951)

[iii) Response to the transducer 3](#_Toc498673952)

[iv) Expected behavior of the biosensor 3](#_Toc498673953)

[2. Creation, multiplication and maintenance of the bioindicator 3](#_Toc498673954)

[3. The search for the optimum bioindicator conditions 6](#_Toc498673955)

[i) Suspended bioindicator in the presence of air 6](#_Toc498673956)

[ii) Bioindicator on surfaces in the presence of air 8](#_Toc498673957)

[iii) Suspended bioindicator in the absence of air 9](#_Toc498673958)

[References 9](#_Toc498673959)

## *In-silico* experiments

A model is required to facilitate a better understanding and optimization of the bioindicator by way of describing the reaction of the receptors on the prophage activating chemicals. The model was created by using Berkley Madonna Version 8.1, developed by R.I. Macey and G.F Oster at the University of California. The describing differential equations were integrated using a Runge Kutta algorithm with an integration interval of dt = 2x10^-3^ h. A reduction in the integration interval has been proven to not have any significant influence on the simulation results. The following model was designed with the aim to take only the main effects into account and use a mathematical structure that is as simple as possible. The basic assumptions of the model were as follows:

### Bioindicator behavior

λ+ and λ- *E.coli* serve as the bioindicators. As the difference in growth behavior between λ+ and λ- is important, the focus will be on the behavior of λ+. Three different subpopulations of the prophage wearing *E. coli* (λ+) strain were postulated. The first subpopulation (*N_R_* in cell L^-1^) is assumed to be resistant against the chemicals and to grow undisturbed (eq. 1).

*dN_R_/dt = µ N_R_*  (1)

*µ = µ_Max_ S/ (k_S_ + S)*  (2)

Here *µ_Max_* and *k_S_* denote the maximum specific growth rate (h^-1^) and the half saturation constant (g L^-1^), respectively. The second subpopulation *N_S_* (cell L^-1^) is sensitive to the chemicals (with a concentration *C* in g L^-1^). *N_S_* grows similarly to *N_R_*; however, a portion of *N_S_* is lost as a result of a transformation into a third subpopulation *N_I_* (cell L^-1^) via a first order kinetics (eq. 3).

*dN_S_/dt = µ N_S_ - k_T_ C N_S_*  (3)

*N_I_* is reduced by lysis and the consequent production of phages. The second process is described via a first order kinetics (eq. 4).

*dN_I_/dt = k_T_ C N_S_ – k_D_ N_I_*  (4)

*k_T_* (L g^-1^ h^-1^) and *k_D_* (h^-1^) are the transformation and the killing constant, respectively.

### Limiting conditions

The substrate consumption *dS/dt* limits the time of the bioindicator response and is described by equation 5.

*dS/dt = - µ (N_R_+N_S_)/ Y_X/S_  - m_E_ (N_R_ + N_S_ )*  (5)

Here are *Y_X/S_* (g g^-1^) and *m_E_* (g cell^-1^ h^-1^) the yield coefficient and the maintenance coefficient, respectively. For the physical meaning of the coefficients, the reader is referred to (Pirt, 1965).

### Response to the transducer

Peltier elements are suggested to serve as thermal transducers. The metabolic heat is dependent on the number of cells *N*, the availability of the substrate *S* as well as the cell specific heat production rate q_t_ (eq. 6).

*Q =∫Q_t_ dt* with *Q_t_ = (q_I_ N_I_ + q_UI_ (N_S_ + N_R_ ))/(S/(k_S_ + S))*  (6)

Pioneering works (Liu et al., 2003; Liu et al., 2005; Mariana Morais et al., 2014) suggest that the cell specific heat production rate of phage producing cells *q_I_* is approx. 30 % higher compared to the cell specific heat production rate of normal cells *q_UI_*.

### Expected behavior of the biosensor

The following parameters (table 1) were used for simulations.

Table 1: The model parameters applied for the simulation of the biosensor behavior.

| Parameter | Value | Dimension | Source |
| --- | --- | --- | --- |
| µ_Max_ | 0.30 | h^-1^ | This work, based on optical density. |
| m_E_ | 1.32 10^-13^ | g cell^-1^ h^-1^ | Estimated from 5.87 mmol ATP g^-1^ h^-1^ (Varma et al., 1993) assuming the EMP pathway and 280 fg per *E. coli* cell (Neidhardt and Umbarger, 1996). |
| k_S_ | 0.01 | g L^-1^ | This work |
| k_T_ | 0.08 | L g^-1^ h^-1^ | This work |
| k_D_ | 1.53 | h^-1^ | Estimated from the typical duration of the lytic cycle (90 min) and assuming a 90% lethality. |
| Y_X/S_ | 4.3·10^13^ | Cells g^-1^ | Estimated from (Heijnen, 1991) assuming a cell specific dry mass of 280 fg (Neidhardt and Umbarger, 1996). |
| *q_I_ /q_UI_* | 1.3 |  | Estimated from data in (Liu et al., 2003; Liu et al., 2005; Mariana Morais et al., 2014) |
| *q_UI_* | 2·10^-13^ | W Cell^-1^ | (Beezer et al., 1974) |

The outcome of the model is discussed in depth in the main document.

## Creation, multiplication and maintenance of the bioindicator

Cultivation conditions

The *E. coli* strains were grown using the LB media formulation according to Lennox (Carl Roth GmbH, Karlsruhe, Germany) either as a liquid or a solid medium at 37 °C. For the plaque assay test, the prophage free strain was pre-incubated in LB medium using MgSO_4_ x 7 H_2_O (0.12 g/L) as well as maltose (2 g/L) for enlarging the phage sensitivity. The double layer technique was applied for estimating the phage titer as plaque forming units mL^-1^.

Calibration curve creation

The growth of the *E. coli* strains was initially observed by the measurement of the optical density at a wavelength of 600 nm. To convert these data into cell numbers, a calibration curve was created. Therefore, a correlation coefficient between the optical density and the amount of living cells (in terms of colony forming units (*cfu*)) was determined. An over-night culture of the *E. coli* WT was refreshed by a 1:100 dilution using with LB medium, cultivated to an OD of 0.477 and diluted to OD 0.384; 0.241; 0.110 and 0.085. Serial dilutions of all 5 subsamples were spread on LB agar plates (triplicates each), incubated overnight and the number of colonies corresponding to the initial OD were counted. The data were correlated using a calibration curve.

Lysogenisation of the *E. coli* WT

The *E. coli* DSM 4230 WT was activated by an overnight cultivation in LB medium to a final OD=0.36 (corresponding to 2x10^8^ cfu/mL). 1 mL *E. coli* WT was infected with the lambda phage (DSM 4499) (with a final titer: 5x10^7^ pfu/mL) corresponding to a MOI (multiplicity of infection) of 0.25. The mixture was incubated for 30 min at 37°C without shaking, serial diluted and plated on LB agar. After 2-day incubation at 37°C and a further incubation at room temperature, single colonies inside the phage plaques were isolated. The isolates were purified several times, checked for lambda phage segregation and sensitivity, and used for further investigations.

Verification of the lysogenization using PCR

Lambda (single and multi) prophage integration was confirmed by PCR following the idea of Powell et al., 1994. In our study, however, with the aim of improving the specificity of the reaction, not only newly designed forward primers but also a modified PCR cycling protocol was employed.

In total, 47 isolates were tested for lysogeny. *E. coli* cells from 500 µL liquid culture were pelleted by centrifugation at 5,700 x g for 10 min. The supernatant was removed and cells were re-suspended in 100 µL sterile water. The cells were disrupted in a microwave at 650 W for 45 sec and the cell debris was removed by centrifugation (5,700 x g, 10 min, 4°C). The DNA containing supernatant was then taken for PCR.

PCR was performed using a Taq PCR Master Mix Kit (Qiagen, Hilden, Germany) in a total volume of 25 µL containing 2 µL of DNA and 100 pmol of each of the three primers. The sequences for the two forward primers were: 5’-TGT CAG GGA TGC AAA ATA GTG TTG-3’ (binding site on *E. coli* genome downstream of the Lambda integration site) and 5’- CTA AGT AGT TGA TTC ATA GTG ACT G-3’ (binding site at the Lambda attP). The reverse primer sequence was taken from Powell et al., 1994. PCR reactions were performed at 95°C for 3 min, followed by 30 cycles (95°C, 50°C and 72°C for 1 min each). PCR amplicons were analyzed on an agarose gel. The amplicon size for lysogenic cultures was 570 bp. A second amplicon of 460 bp was visible when cultures were multilysogenic (together with the 570 bp amplicon).

The PCR results were checked on a 1.5% agarose (LE Agarose; Biozym) gel within 0.5x TAE buffer running for 25 min and 100 V in a MUPID electrophoresis chamber. After staining with ethidium bromide, the results were recorded with a video documentation system (Fig. S1). From the 47 isolates, 26 were found to have one lambda prophage integrated. Three isolates were found to be multilysogenic and eighteen isolates did not carry a lambda prophage. *E. coli* DSM4230 (*lambda*)-47 was selected for further experiments.


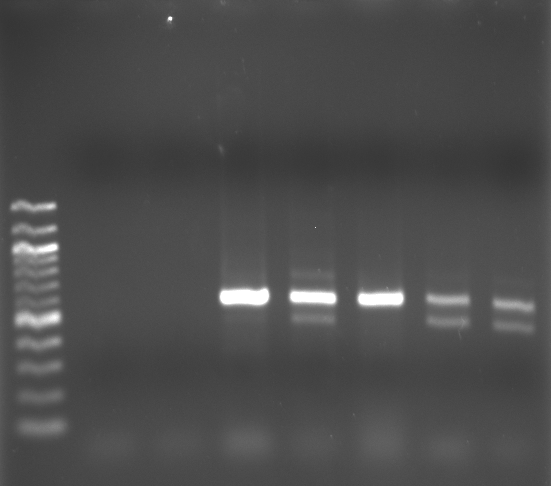


M 1 2 3 4 5 6 7

Figure S1: PCR based integration profiles of the lambda prophage. M denotes the size marker with a 1000 bp and 500 bp dense band; (1) negative control without a template, (2) *E. coli* DSM 4230 WT without a prophage, (3) *E. coli* K124 (lambda) as a positive control, (4)(5)(6)(7) are lambda lysogenic isolates of *E. coli* DSM4230: Isolates (4)(6)(7) are multilysogenic, and isolate (5) is the single lysogenic strain *E. coli* DSM4230 (lambda)-47

## The search for the optimum bioindicator conditions

### Suspended bioindicator in the presence of air

For testing the hypothesis derived from *in-silico* experiments, the bioindicator (*E. coli* (λ+) and (λ-)) were grown aerobically on a thermal transducer in the presence of different amounts of Mitomycin C (Fig. S2).


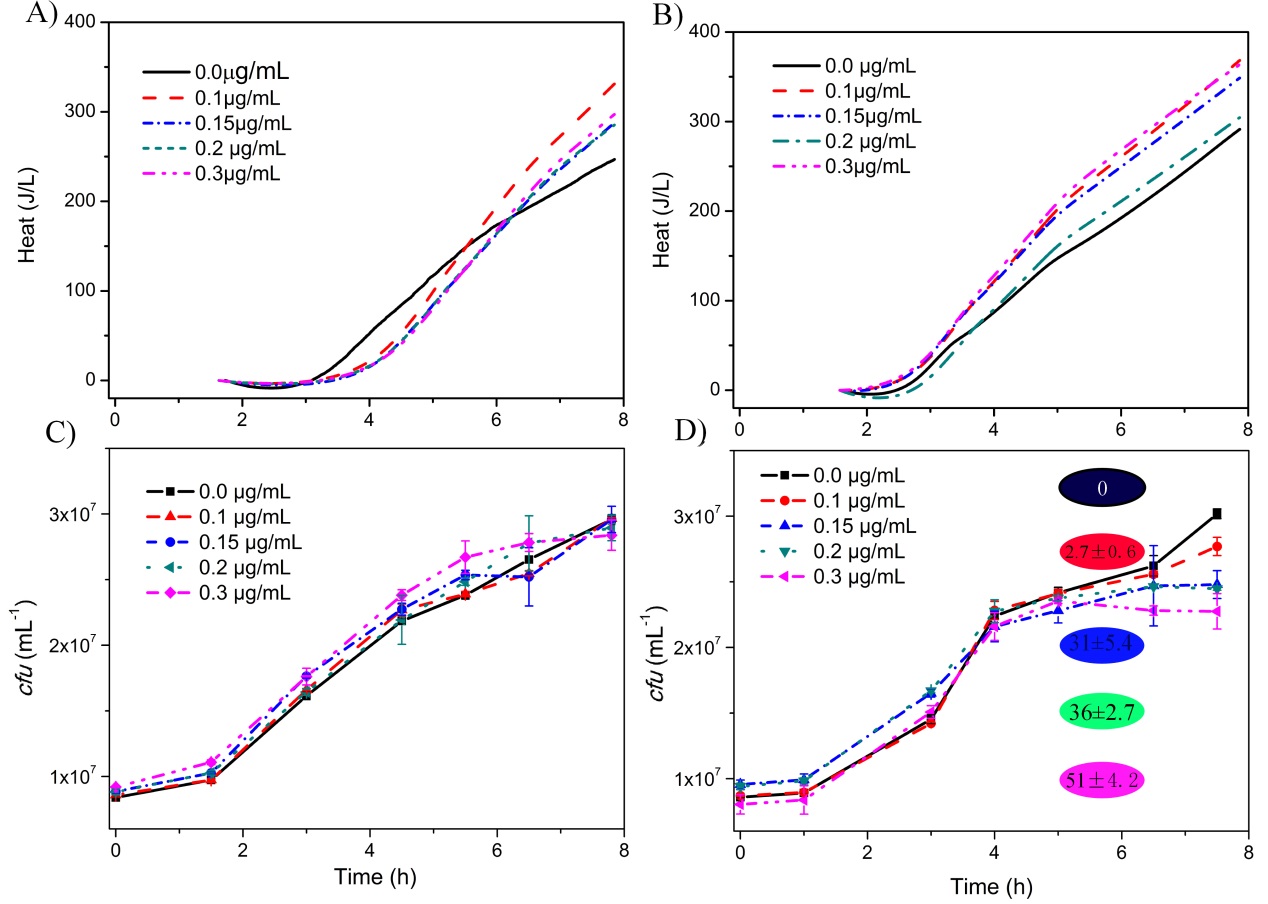


Figure S2: The behavior of the bioindicator (λ- left hand side; λ+ right hand side) in the presence of different amounts of Mitomycin C. A and B show the heat traces and C and D denote the growth traces. The final phage numbers (*10^6^ mL^-1^ given as *pfu* after 8 h) are included in D.

According to the *in-silico* results, there is a much stronger influence of Mitomycin C on λ+ than on the λ- strains regarding the cell numbers. Even the predicted maximum is indicated for 0.2 and 0.3 µg/mL Mitomycin C. The influence on the λ- strains can be explained by the “normal” toxic properties of Mitomycin C. The difference between both traces in growth kinetics is presumed to be the result of the prophage activation. This is confirmed by the kinetics of phage production. Unfortunately, the thermal effect in relation to the measurement variance is too low to be considered as a marker for prophage activation.

A second prophage activating chemical (*cis*-platinum) was tested to find out whether this discouraging result is an exception or the rule. Typical results are depicted in Fig. S3.


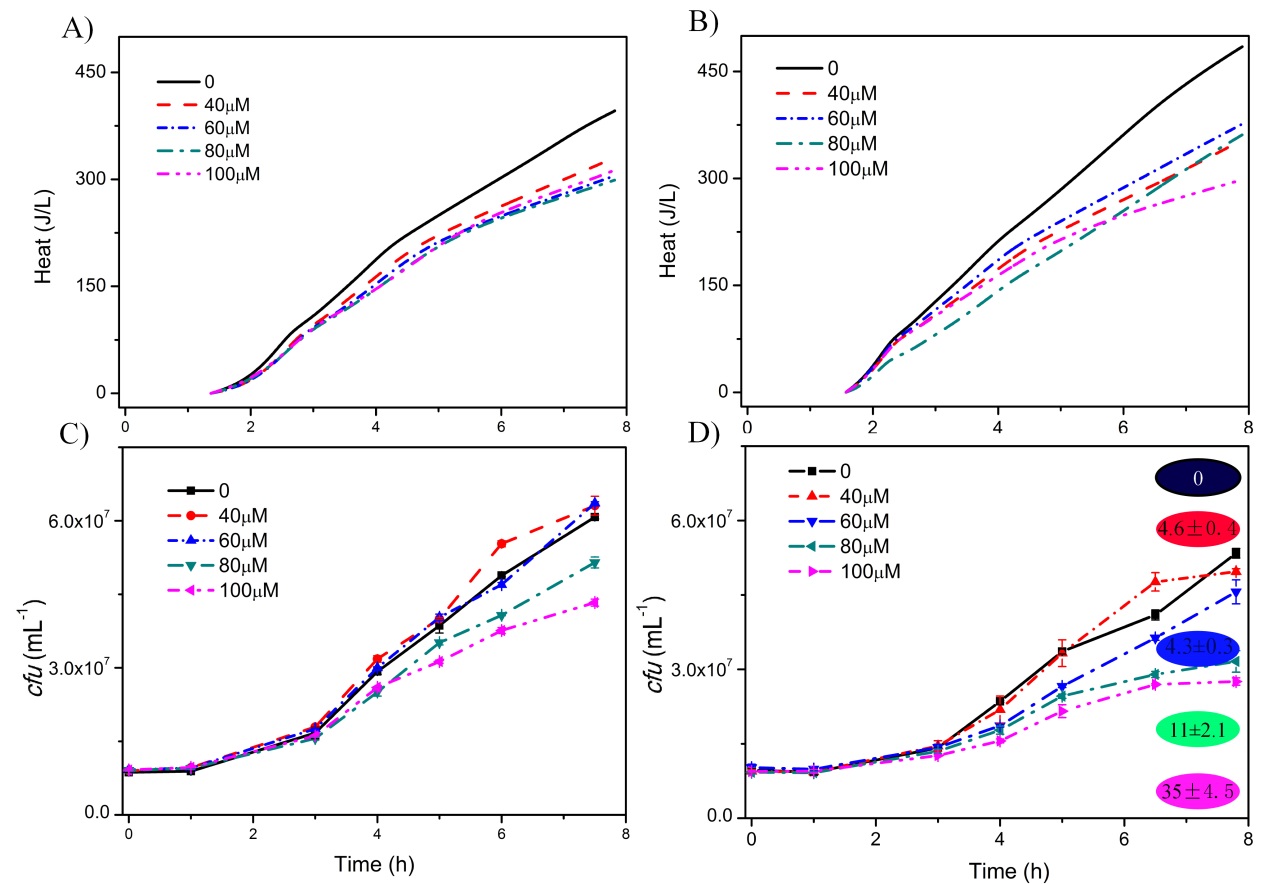


Figure S3: Behavior of the bioindicator (λ- left hand side; λ+ right hand side) in the presence of different amounts of *cis*-platinum C. A and B show the heat traces, and C and D demonstrate the growth traces. The final phage numbers (*10^4^ mL^-1^ given as PFU after 8 h) are included in D.

The bioindicator behavior is similar to that of the example with Mitomycin C.

### Bioindicator on surfaces in the presence of air

This contradictory behavior might have been caused by a metabolic shift from the aerobic to the fermentative-respiratory metabolism of the bioindicator in combination with prophage activation. Such a metabolic shift is known for similar measuring conditions in isothermal calorimetry (Maskow et al., 2014). For testing this thesis, the bioindicators were located on an agar surface to ensure that enough oxygen would be available for a pure aerobic metabolism. The results are shown exemplarily for Mitomycin C as a prophage activating chemical in Fig. S4.


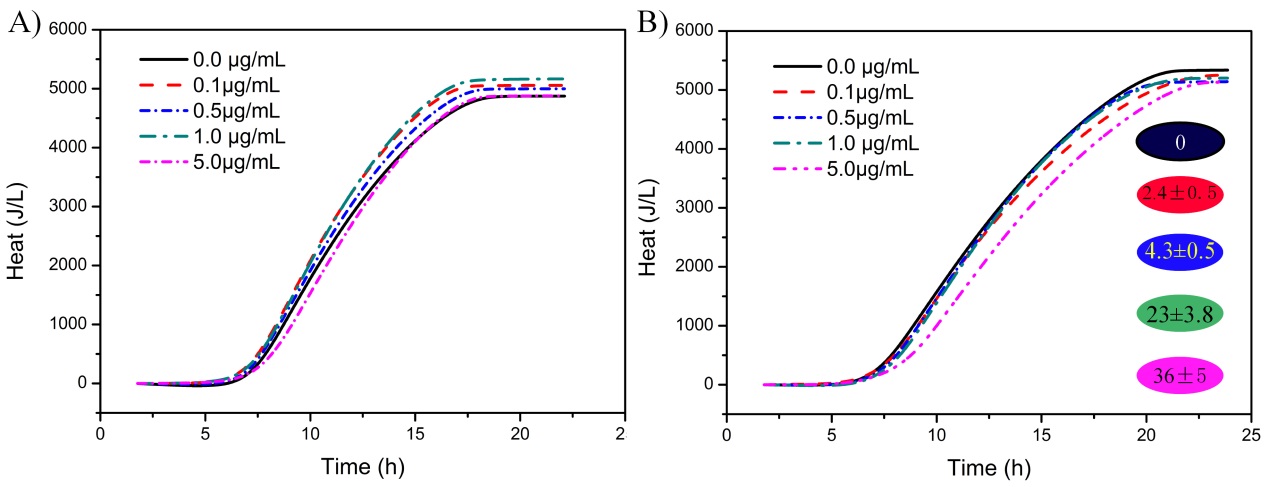


Figure S4: Behavior of the bioindicator (λ- left hand side; λ+ right hand side) grown on agar in the presence of different amounts of Mitomycin C. The final phage numbers (*10^6^ mL^-1^ given as *pfu* after 24 h) are included in circles.

Although the phage test clearly shows the effectiveness of prophage activation by Mitomycin C, the heat trace is hardly affected. A similar result was obtained by using *cis*-platinum for prophage activation. For these reasons, it seems to be unsuitable to operate the bioindicator under aerobic conditions.

### Suspended bioindicator in the absence of air

A good biosensor would require that cell specific heat production rates of infected cells to uninfected cells ${\dot{q}_{i}}/{\dot{q}_{UI}}$ have a ratio of nearly 1 according to the simulation results. This can be achieved when the energy for bioindicator multiplication and maintenance is taken from a fermentative metabolism rather than from an aerobic metabolism. For that reason, the bioindicators were tested under anaerobic conditions. The delivery of oxygen from air was excluded by lamination of the surface of the bioindicator suspension with oil.

## References

Beezer, A.E., Bettelheim, K.A., Newell, R.D., and Stevens, J. (1974) Diagnosis of bacteriuria by flow microcalorimetry: Prilimnary report. Science Tools 21: 13-16.

Heijnen, J.J. (1991) A new thermodynamically based correlation of chemotrophic biomass yields. Antonie van Leeuwenhoek 60: 235-256.

Liu, G.S., Liu, Y., Chen, X.D., Liu, P., Shen, P., and Qu, S. (2003) Study on interaction between T4 phage and Escherichia coli B by microcalorimetric method. Journal of Virological Methods 112: 137-143.

Liu, G.S., Li, M.J., Chen, X.D., Liu, Y., Zhu, J.C., and Shen, P. (2005) Calorimetric study of the metabolic activity of Escherichia coli B infected by T4 phage in restricted medium. . Thermochimica Acta 435: 34-37.

Mariana Morais, F., Buchholz, F., Hartmann, T., Lerchner, J., Neu, T.R., Kiesel, B. et al. (2014) Chip-calorimetric monitoring of biofilm eradication with bacteriophages reveals an unexpected infection-related heat profile. Journal of Thermal Analysis and Calorimetry.

Maskow, T., Mariana Morais, F., Rosa, L.F., Qian, Y.G., and Harnisch, F. (2014) Insufficient oxygen diffusion leads to distortions of microbial growth parameters assessed by isothermal microcalorimetry. RSC Advances 4: 32730 - 32737.

Neidhardt, F.C., and Umbarger, H.E. (1996) Chemical Composition of Escherichia coli.: ASM Press 1996.

Pirt, S.J. (1965) The maintenance energy of bacteria in growing cultures. Proc Roy Soc 163B: 224-231.

Varma, A., Boesch, B.W., and Palsson, B.O. (1993) Stoichiometric interpretation of E coli glucose catabolism under various oxygenation rates. Appl Environ Microb 59: 2465-2473.
